# Developmental and acquired brain injury have opposite effects on finger coordination in children

**DOI:** 10.3389/fnhum.2023.1083304

**Published:** 2023-01-23

**Authors:** Aviva Mimouni-Bloch, Sharon Shaklai, Moran Levin, Moria Ingber, Tanya Karolitsky, Sigal Grunbaum, Jason Friedman

**Affiliations:** ^1^Pediatric Neurology and Development Unit, Loewenstein Rehabilitation Medical Center, Ra’anana, Israel; ^2^Sackler Faculty of Medicine, Tel Aviv University, Tel Aviv-Yafo, Israel; ^3^Department of Child and Youth Rehabilitation, Loewenstein Rehabilitation Medical Center, Ra’anana, Israel; ^4^Department Physical Therapy, Sackler Faculty of Medicine, School of Health Professions, Tel Aviv University, Tel Aviv-Yafo, Israel; ^5^Sagol School of Neuroscience, Tel Aviv University, Tel Aviv-Yafo, Israel

**Keywords:** coordination, uncontrolled manifold hypothesis, children, fingers, force, cerebral palsy, traumatic brain injury, development

## Abstract

The ability to coordinate finger forces to dexterously perform tasks develops in children as they grow older. Following brain injury, either developmental (as in cerebral palsy–CP) or acquired (as in traumatic brain injury—TBI), this developmental trajectory will likely be impaired. In this study, we compared finger coordination in a group of children aged 4–12 with CP and TBI to a group of typically developing children using an isometric pressing task. As expected, deficits were observed in functional tests (Jebsen Taylor test of hand function, Box and Block test) for both groups, and children in both groups performed the pressing task less well than the control group. However, differing results were observed between the CP and TBI groups when using the uncontrolled manifold hypothesis to look at the synergy index. This index measures the relative amount of “good” (does not affect the outcome measure) and “bad” (does affect the outcome measure) variability, where in this case the outcome measure is the total force produced by the fingers. While children with CP were more variable in their performance, their synergy index was not significantly different from typically developing children, suggesting the development of compensatory strategies. In contrast, the children following TBI showed performance that got worse as a function of age (i.e., the older children with TBI performed worse than the younger children with TBI). These differences between the groups may be a result of different areas of brain injury typically observed in CP and TBI, and the different amount of time that has passed since the injury.

## 1. Introduction

In addition to their sensing and expressive roles, our hands and fingers allow us to perform an enormous variety of dexterous tasks (Jones and Lederman, 2006). These tasks are important for many activities of daily living, and many of these tasks involve the coordination of forces in multiple fingers. It is likely that we are able to use our hands in so many different ways due to the large number of degrees of freedom in the hand (Latash, 2012a). This motor abundance (Latash, 2012b), i.e., the ability to take advantage of these degrees of freedom, provides us with flexibility and stability in the way we perform tasks.

The uncontrolled manifold hypothesis (Scholz and Schöner, 1999) has been used to quantify how participants stabilize performance variables by taking advantage of these excess degrees of freedom. Rather than variability in performance necessarily being bad, this technique decomposes the variability into “good” variability, which stabilizes the control variable, and “bad” variability, which causes variability in the control variable—i.e., destabilizes it. For example, if we consider a pressing task with four fingers in the hand where the task requires controlling only the total force, then variance which changes the total force is considered “bad” variability, whereas changes in the force in multiple fingers which does not change the total force (e.g., by negative covariation) is considered “good” variability. In a previous study (Shaklai et al., 2017), we examined how these measures of variability change as a function of age in typically developing children aged 4–12 years old during a pressing task with the fingers. We found that the synergy index, which is a measure of the negative covariation in controlling forces between the fingers, increased approximately linearly from ages 4 to 12, while bad variability decreases over this age range.

In this study, we examined the effects of developmental cerebral palsy (CP) and acquired traumatic brain injury (TBI) brain injury on children’s ability to stabilize control variables over the same age range (4–12 years). We selected these two populations to see the difference in long-term (CP group), and shorter-term (TBI group) brain injury on these measures of variability, as well as standard functional tests. This focus can help us understand the source of motor disorders in hand function in these populations and potentially advise rehabilitation strategies.

Cerebral palsy is a collection of non-progressive permanent disorders that affect movement caused by injury to the developing brain (Patel et al., 2020). The prevalence of CP is approximately 200 per 100,000 live births. Children with CP often show impairment in their hand function (Beckung and Hagberg, 2002; Arner et al., 2008). Previous studies of grasping have shown that children with CP do not develop typical force coordination patterns (Eliasson et al., 1991), and the kinematics of their reaching movements are more segmented and do not show anticipatory shaping of the fingers (Rönnqvist and Rösblad, 2007). Children with CP also show less individuation between the fingers in the paretic hand (McCall et al., 2022). A recent study compared an adult CP group to healthy controls (Kong et al., 2019), and found that the CP group showed smaller maximal forces and higher indices of finger interdependence (enslaving) (i.e., less individuated finger force production in the CP group), while the indices of multi-finger synergies stabilizing total finger forces were not significantly different from those of a control group.

Traumatic brain injury is a result of an injury to the head caused by a physical force resulting in certain symptoms (Thurman, 2016). The prevalence of TBI in children is approximately 180 per 100,000 children, and motor disorders often result from the injury (Yeates et al., 2002). Some studies have reported poor performance on fine motor tasks involving upper-limb speed (Chaplin et al., 1993; Wallen et al., 2001) and dexterity more than 1 year after injury, and deficits in fine motor skills even 7 years post TBI (Emanuelson et al., 1998). Another study showed coordination deficits in children following TBI, plus increased duration of reach-to-grasp movements compared to a control group (Kuhtz-Buschbeck, 2009). In this study, while some recovery was observed after a follow-up period (approximately 8 months following injury) in the TBI group, substantial differences were still observed compared to uninjured children.

In both populations, there has not been an investigation in children examining how indices of multi-finger synergies are affected by brain injury, and how this effect changes with age. In this study, we explored this question. Based on previous studies, we hypothesize that (1) we will see deficits in standard tests (Box and Block, and Jebsen-Taylor) for children with CP and TBI, but they will show improvement with age; (2) Synergy indices for both groups will increase (improve) with age but will be lower than that of typically developing children.

## 2. Materials and methods

### 2.1. Participants

33 participants took part in the experiment, 18 children with moderate to severe TBI based on the Glasgow Coma Scale (Sternbach, 2000) (14 males) and 15 children with CP (9 males). One additional participant was recruited for the TBI group, and eight additional participants were recruited for the CP group but did not participate (1 participant in the TBI group and 3 participants in the CP group were not able to perform the task, and 5 additional subjects for the CP group left the hospital department before they could be tested). General inclusion criteria were children aged 4–12 years old, that were able to understand the task instructions and perform the task and had normal or corrected vision. For the CP group, the participants had developmental motor disorder in at least one of their hands. For the TBI group, the children were in a subacute phase between 6 weeks and 6 months following TBI.

Exclusion criteria included taking sedative medications, other neurological peripheral or orthopedic impairments, complete paralysis or anesthesia in one of the limbs being tested.

Demographic data about the participants can be found in Table 1.

**TABLE 1:** Demographics data about the participants.

Participant	Injury (group)	CP type	Age (Years. Months)	Sex (*M* = male, *F* = female)	Paresis	Ataxia
*TBI1*	TBI		6.11	M	L	N
*TBI2*	TBI		4.11	M	L	B
*TBI3*	TBI		8.9	M	N	N
*TBI4*	TBI		11.0	M	B	N
*TBI5*	TBI		9.5	M	B	B
*TBI6*	TBI		12.0	M	B	B
*TBI7*	TBI		8.4	M	B	B
*TBI8*	TBI		11.11	M	N	N
*TBI9*	TBI		4.10	F	N	B
*TBI10*	TBI		6.2	F	B	B
*TBI11*	TBI		6.2	F	B	N
*TBI12*	TBI		9.1	M	B	B
*TBI13*	TBI		5.10	M	B	N
*TBI14*	TBI		11.11	F	R	N
*TBI15*	TBI		9.10	M	N	N
*TBI16*	TBI		9.4	M	L	N
*TBI17*	TBI		9.4	M	B	N
*TBI18*	TBI		8.7	M	B	N
*CP1*	CP	Spastic quadriparetic	9.10	F	B	N
*CP2*	CP	Spastic diplegic	6.6	F	B	N
*CP3*	CP	Spastic diplegic	7.8	F	B	N
*CP4*	CP	Spastic diplegic	9.0	F	B	B
*CP5*	CP	Spastic triplegic	10.3	F	R	N
*CP6*	CP	Spastic diplegic	6.4	M	N	N
*CP7*	CP	Spastic diplegic	8.4	M	N	N
*CP8*	CP	Spastic diplegic	8.8	M	B	B
*CP9*	CP	Spastic diplegic	10.8	M	B	N
*CP10*	CP	Spastic quadriparetic	6.9	M	B	N
*CP11*	CP	Spastic diplegic	4.10	M	B	N
*CP12*	CP	Spastic diplegic	6.9	M	U	U
*CP13*	CP	Spastic diplegic	9.5	F	N	N
*CP14*	CP	Spastic diplegic	11.7	M	B	N
*CP15*	CP	Spastic quadriparetic	11.9	M	B	N

For injury (group), CP, cerebral palsy; TBI, traumatic brain injury for sex; M, male; F, female for paresis and ataxia; L, left side; R, right side; B, both sides; N, neither side; U, unknown.

The participants provided verbal assent to participate in the experiment, and one of their parents signed an informed consent form prior to participation in the experiment. The experiment was approved and performed according to the guidelines of the Institutional Review Board at the Loewenstein Rehabilitation Medical Center.

The participants in this experiment were compared to a control group of typically developing children, whose data was published previously (Shaklai et al., 2017).

### 2.2. Experimental protocol

The experimental protocol used was the same as that published previously (Shaklai et al., 2017). Briefly, the participants performed two tasks requiring generating isometric force (i.e., without movement) by pressing with the four fingers (index, middle, ring, and little fingers) on piezoelectric force sensors (model 208C01; PCB Piezotronics Inc.). First, they were requested to press as hard as they could for 5 s, to record the maximum voluntary contraction (MVC). This was repeated 3 times with breaks of 30 s between repetitions. Then, they performed a force ramp task, where the total force applied by the four fingers controlled the height of an object on the screen. The participants had to either (a) move a piece of lettuce to track the location of a guinea pig, or (b) move the left half of a rainbow to track the right half of the rainbow. In both cases, the object on the right of the screen (guinea pig or right half of the rainbow) moved up at a constant velocity over 6 s from the bottom to the top of the screen. The participants performed 20 repetitions in total, 10 for each task.

In addition, the participants performed functional tests of upper limb performance: the Jebsen-Taylor hand function test (Jebsen et al., 1969; Taylor et al., 1973), apart from the writing task, with both hands individually, as well as the Box and Block test (Mathiowetz et al., 1985). Both the Jebsen-Taylor hand function test, and the Box and Block test have been shown to be a reliable tool in children with unilateral CP (Araneda et al., 2019; Tofani et al., 2020; Liang et al., 2021).

### 2.3. Data analysis

The data analysis used in this study was the same as that used in our previous study of typically developing children (Shaklai et al., 2017), and the Matlab files used to calculate the quantities are available online (see section “Data availability statement”). For the Jebsen-Taylor hand function test, we compared the total time (in seconds) to complete all the tasks (not including the writing task). For the box and block test, we used the number of blocks the child carried over the partition.

All the force data were collected at 170 Hz, and were filtered using a fourth-order, two-way Butterworth low-pass filter with a cutoff of 4 Hz. The MVC was the maximum sum of the forces produced by the four fingers over the three repetitions. The straight line deviation was calculated as the mean distance from the best-fit line of the sum of forces (using linear regression) from the actual sum of forces. Finger sharing was calculated as the mean percentage of force produced by each of the four fingers. As this was not a main result of the study, the results are presented in the Supplementary material and in Supplementary Figure 1.

Single-trial UCM analysis was used (Scholz et al., 2003; Shaklai et al., 2017). We first detrended the forces by removing the best-fit line found using regression, for each finger. The task in this study was to control the total force:


$$F_{T\,O\,T}=\sum f_{i}$$

Where *f*_*i*_ is the force produced by a single finger. The Jacobian, which is the matrix [1 1 1 1], defines the relationship between the change in finger forces (*df = [df_1_ df_2_ df_3_ df_4_]*) and the change in total force:


$$d\,F_{T\,O\,T}=\left[1 1 1 1\right]\,d\,f$$

For the uncontrolled manifold analysis, we want to know which changes in forces (*df*) do or do not lead to changes in the total force. To find the changes which don’t lead to changes in the total force, we look at the null space of the Jacobian, i.e., solutions for *e*_*i*_ to this equation:


$$0=\left[1 1 1 1\right]\,e_{i}$$

The three solutions to this equation are [−1/2 5/6 −1/6 −1/6]*^T^*, [−1/2 −1/6 5/6 −1/6]*^T^*, and [−1/2 −1/6 −1/6 5/6]*^T^*. We projected the produced forces onto these three null space vectors and took their sum to find the amount of force which does not change the total force, *f*_||_


f||=∑i=13(eiT⋅d⁢f)⁢ei

The remainder of the forces must then be those which do affect the total force *f*_⊥_


$$f_{⊥}=d\,f-f_{\left|\right|}$$

From here, we can calculate the amount of *good* variance, i.e., the variance which does not affect the total force, by squaring *f_||_* and normalizing by its dimension:


$$v_{g\,o\,o\,d}=\sum_{i=1}^{N_{s\,a\,m\,p\,l\,e\,s}}\frac{{\left|f_{\left|\right|}\right|}^{2}}{3\,N_{s\,a\,m\,p\,l\,e\,s}}$$

We define similarly the amount of bad variance:


$$v_{b\,a\,d}=\sum_{i=1}^{N_{s\,a\,m\,p\,l\,e\,s}}\frac{{\left|f_{⊥}\right|}^{2}}{N_{s\,a\,m\,p\,l\,e\,s}}$$

The synergy index *Δv* is the difference between the good and bad variance, normalized by the dimension of the space in which it is calculated:


$$\Delta \,v=\frac{v_{g\,o\,o\,d}-v_{b\,a\,d}}{\left(3\,v_{g\,o\,o\,d}+v_{b\,a\,d}\right)/4}$$

We note that we performed the force analyses for *v*_*good*_ and *v*_*bad*_ in Newton rather than converting them to a percentage of MVC. This was because in some participants (particularly the younger ones), the MVC is relatively low, so small changes in force lead to large changes in the force relative to MVC, leading to outliers. In the main outcome measure, namely the synergy index, the variance is normalized.

### 2.4. Statistical analysis

For the participants in this experiment, we recorded from both the left and right hands, individually. The data for the control group (Shaklai et al., 2017) was only collected for the right, dominant hand (apart from the box and block, and Jebsen Taylor test, which were recorded from both hands).

We used mixed models to analyze the quantities studied here. We used a mixed model as we have both fixed and random effects, described below. We implemented the model using R (R Core Team, 2022), and we used the texreg package (Leifeld, 2013) to generate the tables, and the report package to generate some of the descriptive text (Makowski et al., 2021). To determine which model to use (i.e., which factors to use), we followed a standard procedure (Zuur et al., 2009). In brief, we compared a “beyond optimal” model with only fixed factors (age, group, paretic hand, and their interactions) to a model which also included the random factor of subject and selected the option with the lowest Akaike information criterion (AIC), a measure of model quality. We then compared three options for the fixed factors: age × group × paretic (including all interactions), age × group (including the interaction), age and group (without the interaction), and just age. We selected the model with the lowest Bayesian information criterion (BIC) score, which is another measure of model quality. We note that age was included as a continuous variable. For the selected model, we present here only the factors that had a significant effect and present their sign (i.e., a positive or a negative effect on the outcome variable). Full statistical results are presented in the Supplementary material.

## 3. Results

### 3.1. Maximum voluntary contraction

**FIGURE 1:**
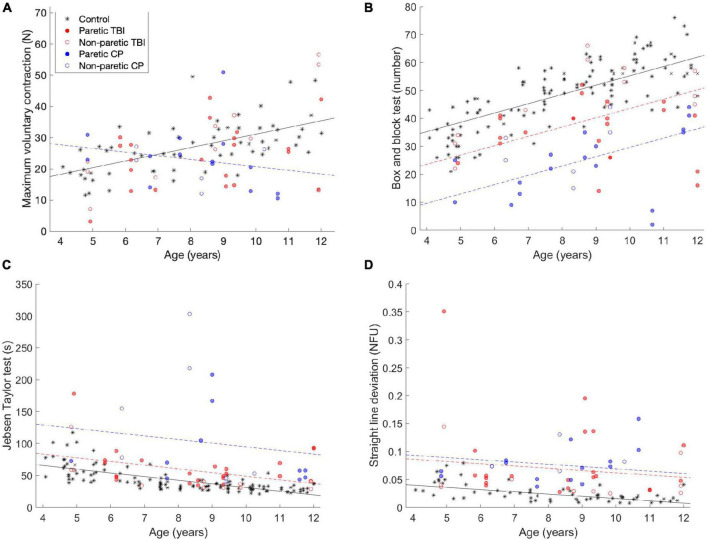
Comparisons of the **(A)** maximum voluntary strength (MVC) data, **(B)** box and block test **(C)** Jebsen-Taylor test of hand function and **(D)** task performance—straight line deviation. The data from this experiment is compared to data from a previous study (the black stars). The black line is the predicted data for the control group, the dashed red line is the predicted data for the TBI group, and the dashed red line is the predicted data for the CP group, according to the tested models. The lines are only drawn when the differences between the groups was significant.

For most of the children, deficits were not observed in terms of the maximum voluntary force produced by the fingers. Figure 1A shows the MVC of the participants, compared to a control group of typically developing children. A main positive effect was observed for age [*t*(103) = 4.72, *p* <.001]—as age increases, so does the MVC. In addition, a negative interaction was found for the CP group and age [*t*(103) = −2.33, *p* = 0.022], as the children with CP get older, they show a larger (negative) difference compared to the control group (see the blue circles in Figure 1). No significant difference was observed for the TBI group.

### 3.2. Functional upper limb tests—Box and Block and Jebsen–Taylor tests

In the Box and Block test, performance improved with age, but was worse for the CP and TBI groups. Figure 1B shows the results of the Box and Block tests. In the Box and Block test, a main positive effect of age was observed [*t*(171) = 8.44, *p* < 0.001]—as the participants got older, they moved more blocks in the minute of the test. A negative main effect was observed for both children with CP [*t*(171) = −9.46, *p* < 0.001] and children with TBI [*t*(171) = −4.98, *p* < 0.001]. These groups performed worse than the control group, but the effect was not mediated by age (i.e., they still improved as a function of age).

For the Jebsen-Taylor hand function tests (Figure 1C), similar results were observed (although they are opposite, because lower scores are better in this test). A main negative effect was observed for age [*t*(105) = 3.91, *p* < 0.001], while main positive effects were observed for the children with CP (*t*(105) = 4.78, *p* < 0.001) and children with TBI [*t*(105) = 5.08, *p* < 0.001].

### 3.3. Performance measure—Straight line deviation

The children improved as a function of age in straight line deviation, which is a measure of performance on the pressing task, while children with CP and TBI performed the task less well. In this task, the participants are asked to apply force with the fingers such that the height of the object they control tracks the second object, which moves linearly up the screen. As a measure of how well they performed the task, we measure how far away the generated force is from a straight line (which would be the ideal performance). The results are shown in Figure 1D. A main negative effect of age was observed [*t*(105) = −2.23, *p* = 0.028]—the older children performed the task more accurately (closer to a straight line). In addition, main positive effects were observed for the CP group [*t*(105) = 4.78, *p* < 0.001] and the TBI group [*t*(105) = 5.08, *p* < 0.001]—the children in both groups performed the task less well, but this was not affected by age.

### 3.4. Uncontrolled manifold (UCM) analysis

**FIGURE 2:**
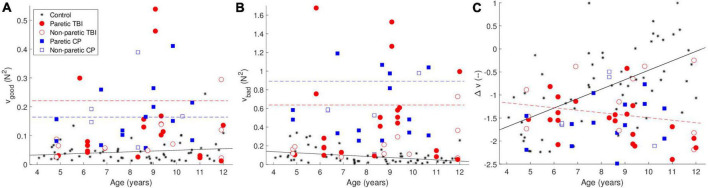
Uncontrolled manifold (UCM) analysis—**(A)** shows the good variance (that which doesn’t affect the outcome variable) **(B)** shows the bad variance (which does affect the outcome variable). **(C)** Shows the synergy index Δv. The black line is the predicted data for the control group, the dashed red line is the predicted data for the TBI group, and the dashed red line is the predicted data for the CP group, according to the tested models. The lines are only drawn when the differences between the groups was significant. The color of the points indicates the group. The black stars are data from typically developing children from a previous study.

The UCM analysis decomposes the variance into “good” and “bad” variance—good variance does not affect the outcome variable (in this case the total force—the height of the object on the screen), and bad variance, which does affect the outcome variable. The synergy index (Δv) is the difference between the amount of good and bad variance values—higher values indicate a larger difference (i.e., relatively more good variance than bad variance). The results are shown in Figure 2.

Both the CP and TBI groups showed greater amounts of both good and bad variance. We observed greater good variance for both the CP [*t*(107) = 5.37, *p* <.001] and TBI [*t*(107) = 4.75, *p* <.001] groups compared to the control group. However, both groups also showed a greater amount of bad variance [CP: *t*(107) = 6.76, *p* <.001; TBI: *t*(107) = 5.69, *p* <.001].

**FIGURE 3:**
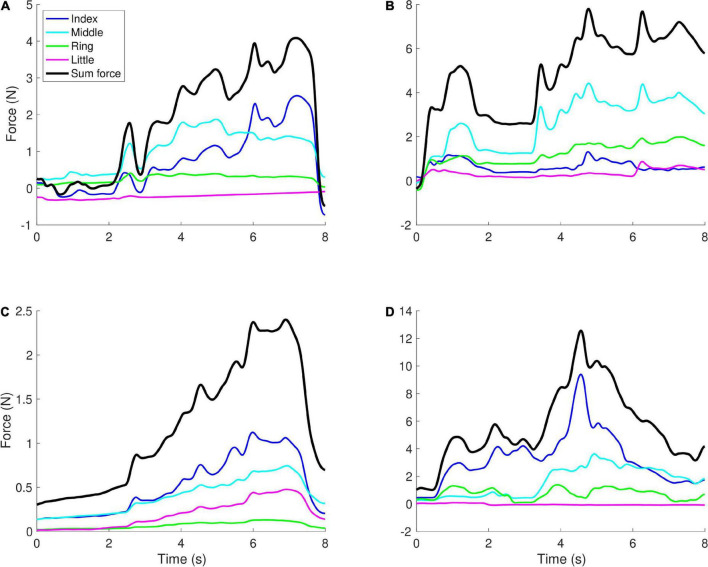
Examples of force profiles. Each graph shows the forces applied by the four fingers in a single trial, with the black line showing the sum of the forces (which controlled the height of the object on the screen). **(A)** A younger child (aged 5) with TBI, Δv = −0.71, **(B)** a younger child (aged 5) with CP, Δv = −1.57, **(C)** an older child (aged 12) with TBI, Δv = −2.15, **(D)** an older child (aged 10) with CP, Δv = −0.77. When there is more positive covariation between the fingers, i.e., in panels **(B,C)**, Δv tends to have lower values (more negative), while when negative covariation is observed, Δv tends to have higher values, i.e., in panels **(A,D)**.

The synergy index Δv showed opposite effects for the CP and TBI groups. We observed a significant positive main effect of age [*t*(103) = 5.79, *p* < 0.001]. While the effect of the CP group was not significant, for the TBI group we observed a significant positive main effect [*t*(105) = 2.63, *p* = 0.010] combined with a significant negative interaction of age and TBI group [*t*(105) = −3.78, *p* < 0.001]. As the children get older, in contrast to the control and CP groups, the synergy index becomes lower (worse) rather than higher. Examples of the forces that lead to these values of Δv are shown in Figure 3.

## 4. Discussion

In this study, we compared the performance of typically developing children and children with CP and TBI aged 4–12 in a finger force pressing task, and in standard functional tests of dexterity. The first hypothesis was supported by the data: functional tests (Jebsen-Taylor and Box and Block test) showed that, as previously reported, performance ameliorated with age, but performance was poorer for children with CP and TBI. The second hypothesis was not supported by the data, rather a more complex relationship was observed. For the main outcome measure, the synergy index, no significant difference was seen for children with CP compared to a control group, and both the control and CP groups improved with age. In contrast, children with TBI showed the opposite pattern—as they got older, their performance became worse.

As expected, the scores for functional tests were lower than those of typically developing children, as has been described previously for the Box and Block and Jebsen-Taylor tests for children with CP (Tomhave et al., 2015; Araneda et al., 2019) and for the Jebsen-Taylor test in children following TBI (Kuhtz-Buschbeck et al., 2003). We note that for the maximum voluntary force (MVC), there was a negative interaction of age and the CP group, while there were no significant differences for the TBI group. This suggests that impairments in maximum finger strength may develop as a function of time (as time since injury is longer in the CP group) and follow a different trajectory to fine motor control of the hand. Additionally, the differences between the findings for the MVC and the functional tests suggest that the impairment in performing these tests is a result of deficits in finger control rather than of finger strength, it may also be due to cognitive effects (Fluss and Lidzba, 2020; Serpa et al., 2021) such as attention or motivation deficits (Marin and Wilkosz, 2005; Salavati et al., 2018) in these populations.

We included whether the hand was paretic as a potential factor in all the statistical tests. However, this was not selected by the algorithm as a factor for explaining any of the quantities. This somewhat surprising result may be related to the observation that while the brain damage may be primarily or exclusively in one hemisphere, both hands are affected (Rich et al., 2017; Burn and Gogola, 2021). In addition, this may be a result of the different brain areas involved in controlling different aspects of movement. Whereas paresis results from injury to the motor areas of the brain or the corticospinal tracts, it may be that the control of the finger force coordination is more reliant on frontal lobe activity. As can be observed in the typically developing children, the factor of age is a strong predictor of the synergy index, which may coincide with the maturation of the frontal lobe development in the ages examined in these studies. The heterogeneity of the participants (see Table 1) and the relatively small sample size may not have allowed significant differences to be observed between the hands.

For children with CP, a significant difference was not observed in terms of the synergy index, which measures how well the children can negatively covariate forces in a way which does not affect the outcome variable. In our previous study (Shaklai et al., 2017), we showed that this index increases as children get older in the range of 4–12 years old. This lack of difference between the control group and the children with CP suggests that with development, children with CP also improve in this ability. In a study of adults with CP using a similar task, a significant difference was also not found for the synergy index, between the adults with CP and a control group (Kong et al., 2019). The differences between the CP and TBI groups may also result from the differences that are typically observed in the brain areas affected in these conditions—children with CP typically are primarily injured in the motor cortex, whereas children with TBI are typically injured in the frontal cortex.

A long-term follow-up of precision grip in children with CP (12 years later) showed significant improvement in most of the measures of performance (Eliasson et al., 2006), suggesting that with development, children with CP show improvements in these measures over time. We note, however, that in this study the children with CP did show differences in other measures of variability. Specifically—they showed significantly greater amounts of good variance, suggesting that overall they are more variable in their performance. The larger amount of good variance may be an indication of compensatory activity used to make up for the deficits in performance of the motor cortex. To test whether this compensatory action is selected somehow by the brain, these participants should be tested in a novel redundant task requiring longer-term learning (e.g., learning to draw novel shapes, while looking at all the joint angles in the arm) to see whether using more good variability in joint angle space to control the end effector is a strategy used across different tasks.

A stark difference, however, was observed between children with CP and children following TBI for the synergy index. For the children following TBI, a decrease in the synergy index with age was observed rather than an increase. This may have to do with changes in plasticity at different ages (Johnston, 2009). This decrease in the synergy index with age is likely due to the increase in bad variability. If we assume that the TBI group are mainly injured in the frontal lobe, and age is also a factor in the maturation of the frontal lobe (Werchan and Amso, 2017), it may be that as age increases in this group, there is less ability for flexibility in frontal lobe control of variability. We note that this differs from the results seen for the functional tests where there was no interaction of age and CP or TBI, it may be that in these tests, the participants can use compensatory strategies (Cirstea and Levin, 2000) that cannot be used in the pressing task.

The opposite trends observed between the CP and TBI groups could also be related to the time since injury. The participants in the TBI group were at the subacute stage, up to 6 months post-injury, thus the plasticity-related processes due to their injuries were likely not completed (Maria et al., 2019), and there may have not been enough time to develop compensatory strategies. In contrast, in the children with CP, it has been many years since the insult, and so the compensatory strategies may have had time to evolve, even for this more complex task. In future research, it would be useful to understand whether the synergy index indeed improves in children following TBI over longer time periods, by performing follow-up experiments on the same children after several years.

There were several limitations of this study. First, we only recorded data from the right hand in the control subjects for most of the measures, so were not able to compare left and right performance between the groups. In addition, due to the nature of the recruitment, the CP and TBI groups were relatively heterogeneous and necessarily do not include the full spectrum of children with CP and TBI (including only those who met the inclusion criteria), which may limit the ability to generalize these findings to these populations in general.

In conclusion, we showed differences in functional tests of hand function, and in a pressing task, between typically developing children, and children with CP and following TBI. In particular, we observed that while for children with CP the synergy index increases as a function of age, in children following TBI, the synergy index decreased as the children got older. These differences may be a result of the differences in brain damage between children with CP (typically damage to motor areas) and children with TBI (typically damage to frontal lobe function), as frontal lobe function is likely responsible for generating the synergies.

## Data availability statement

The datasets presented in this study can be found in online repositories. The names of the repository/repositories and accession number(s) can be found below: https://doi.org/10.6084/m9.figshare.21430227.

## Ethics statement

The studies involving human participants were reviewed and approved by the Institutional Review Board at the Loewenstein Rehabilitation Medical Center. Written informed consent to participate in this study was provided by the participants or their legal guardian/next of kin.

## Author contributions

SS, AM-B, and JF contributed to the conception and design of the study. ML, MI, TK, and SG collected the experimental data. JF performed the statistical analysis and wrote the first draft of the manuscript. All authors contributed to the manuscript revision, read, and approved the submitted version.
