# Supplementary material for: Developmental and acquired brain injury have opposite effects on finger coordination in children
Source: Front Hum Neurosci. 2023 Jan 23;17:1083304. doi: 10.3389/fnhum.2023.1083304 (PMC9899809; doi:10.3389/fnhum.2023.1083304)
Supplement: Supplementary file 1 [file Data_Sheet_1.PDF]

Supplementary Material - Full statistical analysis

For each quantity, we used a mixed model to analyze the data, using R (R Core Team, 2022). The tables are generated automatically using the "texreg" package (Leifeld, 2013), and the descriptive text was automatically generated using the "report" package (Makowski et al., 2021).

For all quantities, standardized parameters were obtained by fitting the model on a standardized version of the dataset. 95% Confidence Intervals (CIs) and p-values were computed using a Wald t-distribution approximation. The results for the first three quantities are summarized in this table, then described in more detail in the following sections:

	Dependent variable:			
	MVC	BB	JT	SLD
	(1)	(2)	(3)	(4)
age	2.146***	3.331***	−5.713***	−0.004*
	(0.454)	(0.395)	(1.217)	(0.002)
groupCP	22.715	−25.615***	63.420***	0.053***
	(11.917)	(2.709)	(9.158)	(0.011)
groupTBI	−5.987	−11.693***	17.666*	0.046***
	(7.865)	(2.348)	(7.065)	(0.009)
age:groupCP	−3.317*			
	(1.421)			
age:groupTBI	0.481			
	(0.902)			
Constant	9.672*	21.922***	88.390***	0.056***
	(3.819)	(3.372)	(10.386)	(0.014)
Observations	111	177	171	111
Log Likelihood	−376.140	−621.934	−787.974	192.606
Akaike Inf. Crit.	768.280	1,255.868	1,587.948	−373.212
Bayesian Inf. Crit.	789.956	1,274.925	1,606.798	−356.954

Note: *p<0.05; **p<0.01; ***p<0.001

1 MVC (MAXIMUM VOLUNTARY CONTRACTION)

We fitted a linear mixed model (estimated using REML and nloptwrap optimizer) to predict MVC with age (formula: MVC age * group). The model included subjectID as random effect (formula: 1 — subjectID). The model's total explanatory power is substantial (conditional R^2 = 0.74) and the part related to the fixed effects alone (marginal R^2) is of 0.29. The model's intercept, corresponding to age = 0, is at 9.67 (95% CI [2.10, 17.25], t(103) = 2.53, p = 0.013). Within this model:

- The effect of age is statistically significant and positive (beta = 2.15, 95% CI [1.25, 3.05], t(103) = 4.72, p $<$ .001; Std. beta = 0.49, 95% CI [0.29, 0.70])
- The effect of group [CP] is statistically non-significant and positive (beta = 22.71, 95% CI [-0.92, 46.35], t(103) = 1.91, p = 0.059; Std. beta = -0.45, 95% CI [-0.98, 0.07])
- The effect of group [TBI] is statistically non-significant and negative (beta = -5.99, 95% CI [-21.59, 9.61], t(103) = -0.76, p = 0.448; Std. beta = -0.21, 95% CI [-0.63, 0.21])
- The interaction effect of group [CP] on age is statistically significant and negative (beta = -3.32, 95% CI [-6.14, -0.50], t(103) = -2.33, p = 0.022; Std. beta = -0.76, 95% CI [-1.41, -0.11])
- The interaction effect of group [TBI] on age is statistically non-significant and positive (beta = 0.48, 95% CI [-1.31, 2.27], t(103) = 0.53, p = 0.595; Std. beta = 0.11, 95% CI [-0.30, 0.52])

2 BOX AND BLOCK

We fitted a linear mixed model (estimated using REML and nloptwrap optimizer) to predict BB with age (formula: BB age + group). The model included subjectID as random effect (formula: 1 — subjectID). The model's total explanatory power is substantial (conditional R^2 = 0.84) and the part related to the fixed effects alone (marginal R^2) is of 0.59. The model's intercept, corresponding to age = 0, is at 21.92 (95% CI [15.27, 28.58], t(171) = 6.50, p $<$ .001). Within this model:

- The effect of age is statistically significant and positive (beta = 3.33, 95% CI [2.55, 4.11], t(171) = 8.44, p $<$ .001; Std. beta = 0.53, 95% CI [0.40, 0.65])
- The effect of group [CP] is statistically significant and negative (beta = -25.62, 95% CI [-30.96, -20.27], t(171) = -9.46, p $<$ .001; Std. beta = -1.76, 95% CI [-2.13, -1.39])
- The effect of group [TBI] is statistically significant and negative (beta = -11.69, 95% CI [-16.33, -7.06], t(171) = -4.98, p $<$ .001; Std. beta = -0.80, 95% CI [-1.12, -0.49])

3 JEBSEN TAYLOR TEST

We fitted a linear mixed model (estimated using REML and nloptwrap optimizer) to predict JT with age (formula: JT age + group). The model included subjectID as random effect (formula: 1 — subjectID). The model's total explanatory power is substantial (conditional R^2 = 0.75) and the part related to the fixed effects alone (marginal R^2) is of 0.36. The model's intercept, corresponding to age = 0, is at 88.39 (95% CI [67.88, 108.90], t(165) = 8.51, p $<$ .001). Within this model:

- The effect of age is statistically significant and negative (beta = -5.71, 95% CI [-8.12, -3.31], t(165) = -4.69, p $<$ .001; Std. beta = -0.36, 95% CI [-0.51, -0.21])
- The effect of group [CP] is statistically significant and positive (beta = 63.42, 95% CI [45.34, 81.50], t(165) = 6.93, p $<$ .001; Std. beta = 1.74, 95% CI [1.24, 2.23])

- The effect of group [TBI] is statistically significant and positive (beta = 17.67, 95% CI [3.72, 31.62], t(165) = 2.50, p = 0.013; Std. beta = 0.48, 95% CI [0.10, 0.87])

4 STRAIGHT LINE DEVIATION

We fitted a linear mixed model (estimated using REML and nloptwrap optimizer) to predict SLD with age (formula: SLD age + group). The model included subjectID as random effect (formula: 1 — subjectID). The model's total explanatory power is substantial (conditional R^2 = 0.56) and the part related to the fixed effects alone (marginal R^2) is of 0.31. The model's intercept, corresponding to age = 0, is at 0.06 (95% CI [0.03, 0.09], t(105) = 3.91, $p < .001$). Within this model:

- The effect of age is statistically significant and negative (beta = -3.75e-03, 95% CI [-7.09e-03, -4.15e-04], t(105) = -2.23, p = 0.028; Std. beta = -0.18, 95% CI [-0.35, -0.02])
- The effect of group [CP] is statistically significant and positive (beta = 0.05, 95% CI [0.03, 0.08], t(105) = 4.78, $p < .001$; Std. beta = 1.16, 95% CI [0.68, 1.64])
- The effect of group [TBI] is statistically significant and positive (beta = 0.05, 95% CI [0.03, 0.06], t(105) = 5.08, $p < .001$; Std. beta = 1.00, 95% CI [0.61, 1.39])

5 FINGER SHARING

The results of the mixed model fitting for the finger sharing are summarized in the following table, then described in more details below. The numbers (1) to (4) correspond to the finger (1=index, 2=middle, 3=ring, 4=little):

	Dependent variable:			
	fingershare			
	linear mixed-effects	*OLS*		*linear mixed-effects*
	(1)	(2)	(3)	(4)
age	−1.491*	0.069	0.544	0.716**
	(0.617)	(0.462)	(0.352)	(0.249)
groupCP	8.991*			
	(4.088)			
groupTBI	7.945*			
	(3.323)			
Constant	44.924***	34.579***	16.027***	5.022*
	(5.288)	(3.920)	(2.982)	(2.119)
Observations	111	111	111	111
R^2		0.0002	0.021	
Adjusted R^2		−0.009	0.012	
Log Likelihood	−438.374			−346.613
Akaike Inf. Crit.	888.747			701.225
Bayesian Inf. Crit.	905.004			712.064
Residual Std. Error (df = 109)		10.913	8.303	
F Statistic (df = 1; 109)		0.022	2.390	
Note:				*p<0.05; **p<0.01; ***p<0.001

5.1 Index finger

We fitted a linear mixed model (estimated using REML and nloptwrap optimizer) to predict fingershare with age (formula: fingershare   age + group). The model included subjectID as random effect (formula: 1 — subjectID). The model's total explanatory power is substantial (conditional R^2 = 0.47) and the part related to the fixed effects alone (marginal R^2) is of 0.13. The model's intercept, corresponding to age = 0, is at 44.92 (95% CI [34.44, 55.41], t(105) = 8.49, p $<$ .001). Within this model:

- The effect of age is statistically significant and negative (beta = -1.49, 95% CI [-2.71, -0.27], t(105) = -2.42, p = 0.017; Std. beta = -0.23, 95% CI [-0.42, -0.04])
- The effect of group [CP] is statistically significant and positive (beta = 8.99, 95% CI [0.89, 17.10], t(105) = 2.20, p = 0.030; Std. beta = 0.61, 95% CI [0.06, 1.17])
- The effect of group [TBI] is statistically significant and positive (beta = 7.95, 95% CI [1.36, 14.53], t(105) = 2.39, p = 0.019; Std. beta = 0.54, 95

5.2 Middle finger

We fitted a linear model (estimated using OLS) to predict fingershare with age (formula: fingershare age). The model explains a statistically not significant and very weak proportion of variance (R^2 = 2.03e-04, F(1, 109) = 0.02, p = 0.882, adj. R^2 = -8.97e-03). The model's intercept, corresponding to age = 0, is at 34.58 (95% CI [26.81, 42.35], t(109) = 8.82, p $<$ .001). Within this model:

- The effect of age is statistically non-significant and positive (beta = 0.07, 95% CI [-0.85, 0.98], t(109) = 0.15, p = 0.882; Std. beta = 0.01, 95% CI [-0.18, 0.20])

Standardized parameters were obtained by fitting the model on a standardized version of the dataset. 95% Confidence Intervals (CIs) and p-values were computed using a Wald t-distribution approximation.

5.3 Ring finger

We fitted a linear model (estimated using OLS) to predict fingershare with age (formula: fingershare age). The model explains a statistically not significant and weak proportion of variance (R^2 = 0.02, F(1, 109) = 2.39, p = 0.125, adj. R^2 = 0.01). The model's intercept, corresponding to age = 0, is at 16.03 (95% CI [10.12, 21.94], t(109) = 5.37, p $<$ .001). Within this model:

- The effect of age is statistically non-significant and positive (beta = 0.54, 95% CI [-0.15, 1.24], t(109) = 1.55, p = 0.125; Std. beta = 0.15, 95% CI [-0.04, 0.33])

5.4 Little finger

We fitted a linear mixed model (estimated using REML and nloptwrap optimizer) to predict fingershare with age (formula: fingershare age). The model included subjectID as random effect (formula: 1 — subjectID). The model's total explanatory power is substantial (conditional R^2 = 0.39) and the part related to the fixed effects alone (marginal R^2) is of 0.08. The model's intercept, corresponding to age = 0, is at 5.02 (95% CI [0.82, 9.22], t(107) = 2.37, p = 0.020). Within this model:

- The effect of age is statistically significant and positive (beta = 0.72, 95% CI [0.22, 1.21], t(107) = 2.87, p = 0.005; Std. beta = 0.27, 95% CI [0.08, 0.46])

6 UNCONTROLLED MANIFOLD (UCM) ANALYSES

	Dependent variable:		
	V_{good}	V_{bad}	Δv
	(1)	(2)	(3)
age	0.004	0.004	0.205***
	(0.006)	(0.021)	(0.035)
groupCP	0.221***	0.891***	0.557
	(0.041)	(0.132)	(0.861)
groupTBI	0.164***	0.637***	1.560**
	(0.034)	(0.112)	(0.594)
age:groupCP			−0.148
			(0.104)
age:groupTBI			−0.259***
			(0.068)
Constant	0.012	0.054	−2.514***
	(0.054)	(0.179)	(0.294)
Observations	113	113	113
Log Likelihood	58.551	−79.388	−110.327
Akaike Inf. Crit.	−105.102	170.775	236.654
Bayesian Inf. Crit.	−88.737	187.139	258.473
Note:	*p<0.05; **p<0.01; ***p<0.001		

6.1 V_{good}

We fitted a linear mixed model (estimated using REML and nloptwrap optimizer) to predict V_{good} with age (formula: V_{good} age + group). The model included subjectID as random effect (formula: 1 — subjectID). The model's total explanatory power is substantial (conditional R^2 = 0.71) and the part related to the fixed effects alone (marginal R^2) is of 0.33. The model's intercept, corresponding to age = 0, is at 0.01 (95% CI [-0.09, 0.12], t(107) = 0.22, p = 0.828). Within this model:

- The effect of age is statistically non-significant and positive (beta = 0.004, 95% CI [-0.00841, 0.02], t(107) = 0.64, p = 0.524; Std. beta = 0.05, 95% CI [-0.11, 0.21])
- The effect of group [CP] is statistically significant and positive (beta = 0.22, 95% CI [0.14, 0.30], t(107) = 5.37, $p < .001$; Std. beta = 1.26, 95% CI [0.79, 1.72])
- The effect of group [TBI] is statistically significant and positive (beta = 0.16, 95% CI [0.10, 0.23], t(107) = 4.75, $p < .001$; Std. beta = 0.93, 95% CI [0.54, 1.32])

6.2 V_{bad}

We fitted a linear mixed model (estimated using REML and nloptwrap optimizer) to predict V_{bad} with age (formula: V_{bad} age * group). The model included subjectID as random effect (formula: 1 — subjectID). The model's total explanatory power is substantial (conditional R^2 = 0.59) and the part related to the fixed effects alone (marginal R^2) is of 0.39. The model's intercept, corresponding to age = 0, is at 0.05 (95% CI [-0.30, 0.41], t(107) = 0.30, p = 0.762). Within this model:

- The effect of age is statistically non-significant and positive (beta = 0.003.85, 95% CI [-0.04, 0.05], t(107) = 0.18, p = 0.854; Std. beta = 0.01, 95% CI [-0.14, 0.17])
- The effect of group [CP] is statistically significant and positive (beta = 0.89, 95% CI [0.63, 1.15], t(107) = 6.76, $p < .001$; Std. beta = 1.47, 95% CI [1.04, 1.90])
- The effect of group [TBI] is statistically significant and positive (beta = 0.64, 95% CI [0.42, 0.86], t(107) = 5.69, $p < .001$; Std. beta = 1.05, 95% CI [0.68, 1.41])

6.3 Δv

We fitted a linear mixed model (estimated using REML and nloptwrap optimizer) to predict δv with age (formula: δv age * group). The model included subjectID as random effect (formula: 1 — subjectID). The model's total explanatory power is substantial (conditional R^2 = 0.71) and the part related to the fixed effects alone (marginal R^2) is of 0.35. The model's intercept, corresponding to age = 0, is at -2.51 (95% CI [-3.10, -1.93], t(105) = -8.56, $p < .001$). Within this model:

- The effect of age is statistically significant and positive (beta = 0.21, 95% CI [0.14, 0.27], t(105) = 5.87, $p < .001$; Std. beta = 0.61, 95% CI [0.40, 0.81])
- The effect of group [CP] is statistically non-significant and positive (beta = 0.56, 95% CI [-1.15, 2.26], t(105) = 0.65, p = 0.519; Std. beta = -0.86, 95% CI [-1.35, -0.37])
- The effect of group [TBI] is statistically significant and positive (beta = 1.56, 95% CI [0.38, 2.74], t(105) = 2.63, p = 0.010; Std. beta = -0.72, 95% CI [-1.14, -0.31])
- The interaction effect of group [CP] on age is statistically non-significant and negative (beta = -0.15, 95% CI [-0.36, 0.06], t(105) = -1.42, p = 0.158; Std. beta = -0.44, 95% CI [-1.05, 0.17])
- The interaction effect of group [TBI] on age is statistically significant and negative (beta = -0.26, 95% CI [-0.39, -0.12], t(105) = -3.79, $p < .001$; Std. beta = -0.76, 95% CI [-1.16, -0.36])

REFERENCES

Leifeld, P. (2013). texreg: Conversion of statistical model output in R to LaTeX and HTML tables. *Journal of Statistical Software* 55, 1–24

Makowski, D., Ben-Shachar, M. S., Patil, I., and Lüdecke, D. (2021). Automated results reporting as a practical tool to improve reproducibility and methodological best practices adoption. *CRAN*

R Core Team (2022). *R: A Language and Environment for Statistical Computing*. R Foundation for Statistical Computing, Vienna, Austria
