# Supplementary material for: Developmental and acquired brain injury have opposite effects on finger coordination in children
Source: Front Hum Neurosci. 2023 Jan 23;17:1083304. doi: 10.3389/fnhum.2023.1083304 (PMC9899809; doi:10.3389/fnhum.2023.1083304)
Supplement: Supplementary file 2 [file Data_Sheet_2.PDF]

Supplementary Material – Finger sharing

Supplementary Material – Finger sharing

1 Finger Sharing

Finger sharing represents the mean percentage that each finger contributes to the overall force, see Figure 2. No significant differences we found for the middle and ring fingers (including for age), and so we focus on the results for the index and little fingers only. The index finger contributes most of the force, and this is observed to decrease with age (t(105)=-2.42, p = 0.017). In addition, in both the CP group (squares in the figure; t(105)=2.20, p=0.03) and the TBI group (circles in the figure; t(105)=2.39, p=0.019) there were positive significant effects, i.e., these groups used the index finger more than the control group. For the little finger, a significant positive effect was seen for age (t(107)=2.31, p = 0.023), i.e., more use of the little finger in older children, but there was no significant difference in the use of the finger between groups.

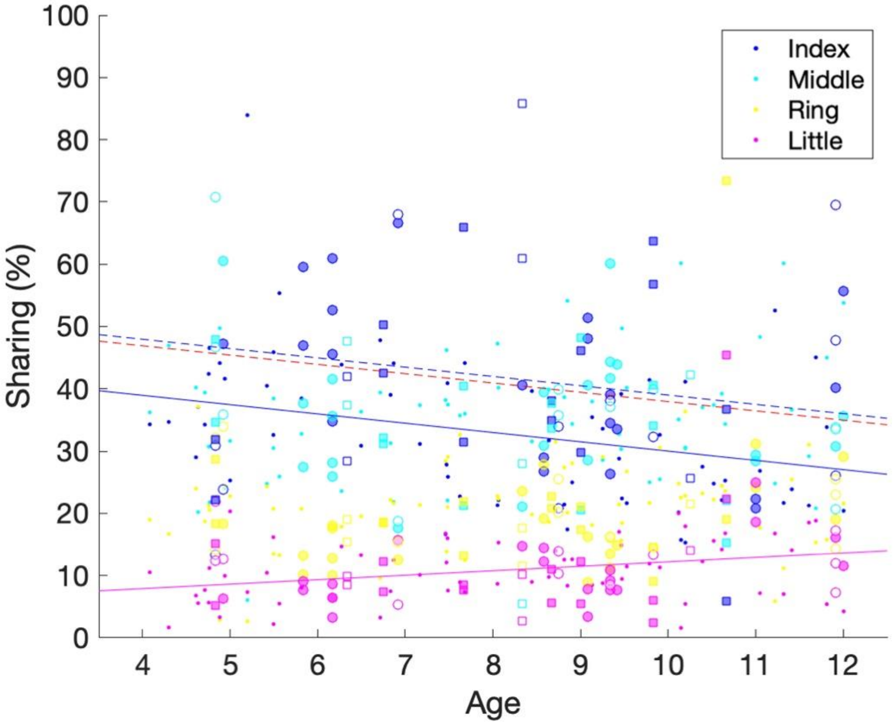

Supplementary Figure 1. Finger sharing – the mean proportion of the total force produced by each of the four fingers. The solid circles indicate a paretic hand of the TBI group, the unfilled circles a non-paretic hand for the TBI group. The solid squares indicate a paretic hand in the CP group, the unfilled squares indicate a non-paretic hand in the CP group. The data for the TBI and CP groups are from both hands, for the control group only data from the right hand is shown. The solid blue line is the model prediction for the index finger for the control group, the dashed red line is the predicted index finger share for the TBI group, and the dashed red line is the predicted index finger share for the CP group. The pink line is the predicted share for the little fingers.
